# Identification and functional analysis of circular RNAs in intervertebral disc lesion tissues from spinal tuberculosis patients

**DOI:** 10.3389/fphys.2025.1650380

**Published:** 2025-09-26

**Authors:** Linan Wang, Xingyu Duan, Yuliang Qu, Zhiyun Shi, Xu Zhang, Zongqiang Yang, Yingchao Gao, Ningkui Niu

**Affiliations:** ^1^ Department of Orthopedics, General Hospital of Ningxia Medical University, Yinchuan, Ningxia, China; ^2^ The First Clinical Medical College of Ningxia Medical University, Yinchuan, Ningxia, China; ^3^ Ningxia Medical University, Yinchuan, Ningxia, China; ^4^ Medical Experiment Center, General Hospital of Ningxia Medical University, Yinchuan, Ningxia, China; ^5^ Department of Surgery Laboratory, Institute of Medical Sciences, General Hospital of Ningxia Medical University, Yinchuan, Ningxia, China; ^6^ Department of Orthopedics, No. 942 Hospital of the Joint Logistics Support Force of the People’s Liberation Army, Yinchuan, Ningxia, China; ^7^ Research Center for Prevention and Control of Bone and Joint Tuberculosis, General Hospital of Ningxia Medical University, Yinchuan, Ningxia, China

**Keywords:** spinal tuberculosis, circular RNA, intervertebral disc, gene chip, pathogenesis

## Abstract

**Background:**

Spinal tuberculosis (STB) is characterized by an insidious onset, nonspecific clinical manifestations, and diagnostic challenges in early stages. Circular RNAs (circRNAs), a class of stable single-stranded endogenous RNAs with covalently closed-loop structures, play crucial regulatory roles in various biological processes. However, their involvement in STB pathogenesis remains largely unexplored.

**Methods:**

We collected intervertebral disc tissue specimens and peripheral blood samples from STB patients. CircRNAs sequencing was performed on three representative lesion tissues, followed by comprehensive screening and validation in both tissue and blood samples to elucidate the functional mechanisms of circRNAs in STB.

**Results:**

Chip sequencing indicated that a total of 1,396 circRNAs were differentially expressed (fold change >2, *P* < 0.05). Among them, 757 circRNAs were upregulated, while 639 were downregulated. GO analysis of the parental genes of these differentially expressed circRNAs demonstrated that they were predominantly involved in cellular protein catabolic processes, GTPase binding, covalent chromatin modification, histone modification, and other processes. KEGG pathway analysis revealed that these genes were mainly enriched in signal pathways such as protein processing in the endoplasmic reticulum, apoptosis, and proteolysis. Validation in peripheral blood samples showed that the expressions of hsa_circ_0001021, hsa_circ_0043898, and hsa_circ_0093669 were significantly higher in the preoperative group than in the control group (*P* < 0.05). Moreover, the expression levels of these circRNAs decreased at 6 months and 1 year postoperatively compared to the preoperative levels. ROC curve analysis suggested that these circRNAs could serve as promising diagnostic biomarkers for STB in academic research scenarios.

**Conclusion:**

The differentially expressed circRNAs identified in intervertebral disc lesions of STB patients demonstrate significant involvement in disease pathogenesis, positioning them as promising candidates for both diagnostic and therapeutic targeting. Specifically, hsa_circ_0001021, hsa_circ_0043898, and hsa_circ_0093669 exhibit robust potential as peripheral blood-based diagnostic biomarkers for STB. When combined with conventional inflammatory markers ESR and CRP, these circRNAs significantly enhance diagnostic accuracy. Furthermore, longitudinal monitoring revealed their unique value in postoperative therapeutic evaluation, with expression levels showing strong correlation with treatment response.

## Background

According to the Global Tuberculosis Report, tuberculosis remains a chronic infectious disease that poses significant threats to global health and continues to be prioritized by the World Health Organization (WHO) for targeted control measures. Osteoarticular tuberculosis represents the most prevalent form of extrapulmonary TB, with spinal tuberculosis (STB) constituting approximately 50% of all skeletal tuberculosis cases. The pathogenesis of STB involves hematogenous dissemination of *Mycobacterium tuberculosis* (MTB) through paravertebral end arteries or valveless venous plexuses (Ali et al., 2019; Garg and Somvanshi, 2011). Primary lesions typically originate in the anterior vertebral column, with vertebral body involvement observed in 99% of cases, compared to only 1% involving posterior spinal elements. This condition demonstrates predilection for pediatric and young adult populations. Clinically, STB manifests with an insidious onset characterized by nonspecific symptoms during early stages, with merely 20%–30% of patients presenting detectable clinical manifestations at initial presentation (Kilinc et al., 2023). Untreated STB may progress to severe neurological complications including spinal cord compression, vertebral instability, progressive kyphotic deformity, and permanent paralysis (Pang et al., 2019; Jain et al., 2020; Yang et al., 2021). Given this clinical trajectory, implementation of early diagnostic protocols - the cornerstone of effective management - followed by prompt therapeutic intervention and rehabilitation is strongly advocated in contemporary practice. STB frequently evades early detection due to the absence of specific diagnostic biomarkers. Current diagnosis relies on comprehensive evaluation of medical history, clinical manifestations, imaging findings, and laboratory tests, often resulting in missed diagnoses, misdiagnoses, and even inappropriate treatments (Pang et al., 2019; Jain et al., 2020; Yang et al., 2021). This clinical reality underscores the urgent need to elucidate STB pathogenesis and identify reliable biomarkers to facilitate early diagnosis and intervention. Circular RNAs (circRNAs) represent a novel class of endogenous RNAs formed through back-splicing of pre-mRNA, primarily derived from exons (Pang et al., 2019; Jain et al., 2020; Yang et al., 2021). Characterized by covalently closed loop structures lacking 5′caps and 3′poly (A) tails, circRNAs exhibit remarkable resistance to exonuclease-mediated degradation, conferring exceptional molecular stability (Chen, 2020; Misir et al., 2022). While predominantly localized in the cytoplasm with minor nuclear presence (Chen, 2020; Guo et al., 2020; Heyde et al., 2021), circRNAs function as potent post-transcriptional regulators through their microRNA (miRNA) “sponge” activity - competitively binding miRNAs to attenuate their suppressive effects on target genes (Zheng L. et al., 2022). These distinctive molecular properties, combined with disease-specific expression patterns, position circRNAs as ideal diagnostic biomarkers (Zhang et al., 2020; Chen et al., 2021; Misir et al., 2022). Emerging research highlights circRNAs’ diagnostic and prognostic potential in pulmonary tuberculosis (Qian et al., 2018; Li et al., 2023). In MTB-infected individuals, circRNAs modulate immunocompetent cells (B cells, T cells, macrophages) to influence bacterial clearance. Moreover, differentially expressed circRNAs in peripheral blood show tremendous promise as diagnostic markers and prognostic indicators for tuberculosis.

This study employed circRNA microarray technology to conduct a comprehensive transcriptomic analysis of intervertebral disc lesion tissues from STB patients, systematically screening differentially expressed circRNAs. Through bioinformatics interrogation, we performed Gene Ontology (GO) analysis (categorized by biological process, cellular component, and molecular function) and Kyoto Encyclopedia of Genes and Genomes (KEGG) pathway analysis to elucidate their potential roles in STB pathogenesis. Three stably differentially expressed circRNAs (hsa_circ_0001021, hsa_circ_0043898, and hsa_circ_0093669) were identified and validated in STB peripheral blood. Their diagnostic utility was evaluated using receiver operating characteristic (ROC) curve analysis. Furthermore, we collected serial peripheral blood samples from patients undergoing radical lesion debridement followed by ultra-short-course chemotherapy at three critical timepoints: admission, 6-month postoperative, and 1-year follow-up. Quantitative reverse transcription PCR (qRT-PCR) was employed to track expression dynamics of these circRNAs, with comparisons made against both healthy controls and baseline patient levels to assess their diagnostic and prognostic value. This investigation provides a robust theoretical foundation for developing circRNA-based microarrays to facilitate early detection, accurate diagnosis, prognostic evaluation, and mechanistic exploration of STB pathogenesis.

## Methods

### Ethical approval and patient consent

All study procedures were approved by the Institutional Review Board/Ethics Committee of General Hospital of Ningxia Medical University (Approval No: KYLL-2022-0133) in accordance with the Declaration of Helsinki principles. Written informed consent was obtained from all participating patients or their legal guardians prior to sample collection and data acquisition.

### Sample collection

Inclusion criteria for the observation group: Based on at least one of the following criteria for diagnosing spinal tuberculosis: a positive culture of *M. tuberculosis* obtained from biopsy or surgical specimens, positive acid-fast staining observed in histopathological analysis, detection of *M. tuberculosis* through metagenomic next-generation sequencing (mNGS), or a positive result from the Xpert MTB/RIF test. Patients with STB who were diagnosed based on clinical manifestations, imaging, laboratory tests, and histopathological examination, and had surgical indications and underwent surgery at the General Hospital of Ningxia Medical University were included in the study. Exclusion criteria: patients with other sites of tuberculosis, immune deficiency diseases, tumors, metabolic diseases, etc.

Inclusion criteria for the control group: patients with lumbar intervertebral disc degeneration who were diagnosed based on medical history and imaging, had surgical indications, and underwent surgery at the General Hospital of Ningxia Medical University. Exclusion criteria: patients with tuberculosis combined with other infectious diseases, tumors, trauma, immune deficiency diseases, metabolic diseases, etc.

According to the inclusion and exclusion criteria, 35 patients with STB who underwent surgery from September 2019 to December 2020 were selected, and 35 patients with lumbar intervertebral disc degeneration were included as the control group. Three specimens were randomly selected from the observation group, including 1 case of L1-2 tuberculosis and 2 cases of L2-3 tuberculosis. Three specimens were randomly selected from the control group, including 1 case of L3-4 intervertebral disc (lumbar spinal stenosis) and 2 cases of L4-5 intervertebral disc (lumbar intervertebral disc protrusion), as shown in Table 1. Peripheral blood samples were collected from the observation group patients before surgery (35 cases), at 6 months after surgery (28 cases), and at 1 year after surgery (16 cases), and were divided into groups A, B, and C, respectively. Peripheral blood samples were also collected from 35 patients in the control group. All cases were approved by the Ethics Committee of Ningxia Medical University and informed consent was obtained from the patients.

**TABLE 1:** General information of patients.

Project	Observation group	Control group
Number of cases, n	3	3
Gender (male/female), n	1/2	2/1
Age/years, ( $\bar{x}$ ± *s*)	32.3 ± 4.6	54 ± 1.7

### RNA extraction and sequencing

Total RNA was extracted from six intervertebral disc specimens (three from STB lesions and three from surgical disc herniation cases) and peripheral blood samples using Trizol reagent. To enrich circular RNAs, total RNA was treated with RNase R to degrade linear RNAs. The enriched circRNAs were then hybridized to Arraystar Human circRNA Array V2 (8 × 15K, Arraystar). Following hybridization and washing, the arrays were scanned using an Agilent Scanner G2505C. The scanned images were processed and analyzed with Agilent Feature Extraction software (version 11.0.1.1) to obtain circRNA expression profiles for both experimental and control groups. This study cannot identify new circRNAs during gene sequencing; it can only identify currently known circRNAs. These circRNAs IDs can be found in circBase (http://www.circbase.org/) or other literature. Differentially expressed circRNAs between groups were identified using the following criteria: fold change (FC) ≥ 2 or ≤ −2 with *P* < 0.05 as determined by Student’s t-test.

### Bioinformatics analysis of circRNAs

Based on the chip screening results, the parent genes of significantly differentially expressed upregulated and downregulated circRNAs were selected and subjected to GO analysis (categorized into three ontologies, including biological processes, cellular components, and molecular functions) and KEGG pathway analysis using the Metascape online database (https://metascape.org/gp/index.html#/main/step1) (Zhou et al., 2019).

### Validation of differentially expressed circRNAs by qRT-PCR

Due to the relatively low expression level of circRNAs and their special circular structure, the 15 circRNAs with the most significant differential upregulation and downregulation were selected and 35 circRNAs related to inflammation were identified. Using the CircPrimer2.0 software, the basic information and base sequences of the target circRNAs were obtained and cross-ring primers were designed. Glyceraldehyde-3-phosphate dehydrogenase (GAPDH) was used as the internal control. The relative expression levels were calculated using 2^−ΔΔCt^ (ΔCt = CT of the target gene - CT of the internal control, ΔΔCt = ΔCT of the target gene - ΔCt of the average value of the control group). FC = 2^-ΔΔCt^ (ΔCt = CT of the target gene - CT of the internal control, ΔΔCt = ΔCT of the target gene - ΔCt of the control group). The primers are shown in Table 2.

**TABLE 2:** Primer sequences used for qRT-PCR.

Name of gene	Forward/Reverse	Primer sequence (5'−3 ′)	Product (bp)
hsa_circ_0001021	F	CCCAAAACGCACAGTGTACC	161
	R	TGGTGGTGGGGATGAAGAGT	
hsa_circ_0043898	F	AGCTTCGTGTCCAACCTGTT	97
	R	GAATGGCAGGACACAACAGC	
hsa_circ_0093669	F	TGGGGAGTGTCTAGCCATGA	86
	R	CTGATGCTGACGGAGGACTC	
hsa_circ_0111766	F	CACTCTGTCTGGGCTGGATG	193
	R	TGTCCTCCAGTAATCCTCACGA	
GAPDH	F	CAGGAGGCATTGCTGATGAT	138
	R	GAAGGCTGGGGCTCATTT	

### Prediction of circRNA target miRNAs

CircRNAs can act as a molecular “sponge” for miRNA, regulating the function of miRNA by binding to multiple miRNA response elements (MREs). It plays a crucial role in post-transcriptional gene regulation involving miRNAs. To study the functional annotation of differentially expressed circRNAs, based on the chip screening results, the top ten significantly upregulated and top ten significantly downregulated circRNAs were selected. The miRanda and TargetScan algorithms were used to predict possible interactions between circRNAs and related miRNAs. The top five miRNAs with the best matching values for each circRNA were selected, and a two-dimensional interaction graph of circRNA and miRNA was obtained.

### Statistical analysis

During the experiment, measurement data were expressed as $\bar{x}$ ± s, and count data were expressed as percentages (%). The Shapiro-Wilk test was used to test the normality of the measurement data, and the Brown-Forsythe test was used to test the homogeneity of variance. If the data followed a normal distribution and the variances were homogeneous, the independent sample t-test was used for comparison between the two groups of measurement data, and one-way ANOVA was used for comparisons among multiple groups. The count data were tested using the chi-square test. Non-parametric tests were used if the data did not follow a normal distribution. Statistical analysis and graphing were performed using SPSS 26.0 and GraphPad 8.0.1 software. Bivariate Spearman correlation analysis was used to examine the correlation between circRNA and the clinical data of the observation group. The ROC curve was drawn using MedCalc 15.2 software, and the area under the ROC curve (AUC) was calculated to evaluate the diagnostic performance of circRNA. A difference was considered statistically significant if *P* < 0.05, and circRNA had diagnostic value for STB if the AUC ≥0.6.

## Results

### Patient general information

Among the 35 patients with STB in the observation group, there were 20 males and 15 females. The youngest and oldest ages were 22 years and 81 years respectively, with an average age of 51.24 ± 14.35 years. The disease duration ranged from 3 weeks to 13 months, with an average duration of 4.52 ± 2.57 months. There were 14 cases of lesions in the thoracic vertebrae, 5 cases in the thoracic and lumbar vertebrae, 15 cases in the lumbar vertebrae, and 1 case in the lumbar-sacral segment. Secondary paravertebral and psoas muscle abscesses were present in 20 and 3 cases respectively, and 1 case was found to have abscesses in both the paravertebral area and the spinal canal. Among the 35 patients with intervertebral disc degeneration included in the study, there were 23 males and 12 females. The youngest and oldest ages were 35 years and 76 years respectively, with an average age of 55.18 ± 11.35 years. There was no statistically significant difference in age and gender between the observation group and the control group (*P* > 0.05). In the early stages of STB patients often do not experience significant lower back pain. From the onset of the disease to the time of hospital visit and diagnosis, 80% of patients have a disease course exceeding 14 days. Most patients do not have elevated white blood cell counts, but there is a mild increase in neutrophils and lymphocytes. However, in the early stages of the disease, the majority of patients have elevated erythrocyte sedimentation rate (ESR, 94.3%) and C-reactive protein (CRP, 94.3%). Some patients will see their inflammatory indicators return to normal after anti-tuberculosis drug treatment. We have found that the T-SPOT test in peripheral blood has a certain rate of false positives, and we need to make a comprehensive judgment based on the patient’s medical history, symptoms, imaging, and other test indicators. We sent the diseased tissue removed during surgery for Xpert MTB/RIF testing and found that it has high specificity and sensitivity. Referring to the results in combination with mNGS can significantly improve the diagnosis rate of spinal tuberculosis, as shown in Tables 3, 4.

**TABLE 3:** Comparison of age and gender between the two groups of patients.

Group	n	Mean age (years, ( $\bar{x}$ ±*s*))	Sex [n, (%)]	
			Male	Female
Observation Group	35	51.24 ± 14.35	20 (57.14)	15 (42.86)
Control Group	35	55.18 ± 11.35	23 (65.71)	12 (34.29)
Test Statistic	—	t = 1.465	χ2 = 0.543	
*P*-value	—	0.222	0.461	

**TABLE 4:** Basic information of patients in the observation group.

Parameters	No. of Cases (%)
Course (d)	
>14	28 (80%)
≤14	7 (20%)
NEUT%	
Normal	25 (71.4%)
Abnormal	10 (28.6%)
MXD%	
Normal	23 (65.7%)
Abnormal	12 (34.3%)
ESR (mm/h)	
Normal	33 (94.3%)
Abnormal	2 (5.7%)
CRP (mg/L)	
Normal	33 (94.3%)
Abnormal	2 (5.7%)
T-SPOT positive	
Yes	32 (91.4%)
No	3 (8.6%)
Xpert MTB/RIF	
Positive	34 (97.1%)
Negative	1 (2.9%)
mNGS	
Positive	35 (100%)
Negative/Other	0
Imaging diagnosis positive	
Yes	30 (85.7%)
No	5 (14.3%)
Pathology positive	
Yes	24 (68.6%)
No	11 (31.4%)
Acid-fast staining positive	
Yes	10 (28.6%)
No	25 (71.4%)
Bacteriological positive	
Yes	12 (34.3%)
No	23 (65.7%)

### Gene chip screening of differentially expressed circRNAs in the diseased intervertebral disc tissues of patients with STB

**FIGURE 1:**
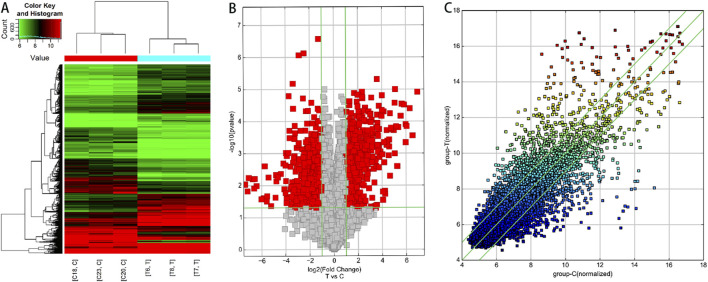
Analysis of differentially expressed circRNA profiles in lesioned intervertebral disc tissues of STB patients compared to degenerative disc tissues. **(A)** Hierarchical clustering analysis. Red indicates upregulated circRNAs (FC > 2), while green represents downregulated circRNAs (FC < −2). C: Degenerative disc tissue samples; T: STB-affected disc tissue samples. **(B)** Volcano plot. Red dots denote differentially expressed circRNAs (FC ≥ 2, *P* < 0.05) in STB-affected discs compared to degenerative discs. Green vertical baselines demarcate FC > 2 or FC < −2, and the horizontal green baseline indicates *P* < 0.05. The right side of the plot highlights circRNAs upregulated in STB discs, while the left side shows downregulated circRNAs. **(C)** Scatter plot. This plot visually displays circRNAs with FC > 2 and *P* < 0.05 in STB-affected discs versus degenerative discs. The X-axis and Y-axis represent the mean normalized signal values of degenerative discs and STB-affected discs, respectively. The central green line (FC = 1) indicates no difference between groups. circRNAs above the upper green line (FC > 2) or below the lower green line (FC < −2) are considered significantly differentially expressed.

Based on the results of circRNA chip analysis, significant differences in circRNA expression were observed between the observation group and the control group. A total of 1,396 differentially expressed circRNAs (|FC| ≥ 2, *P* < 0.05) were identified, with 757 upregulated and 639 downregulated. To visualize the results intuitively, hierarchical clustering heatmap, scatter plot, and volcano plot were generated based on the chip data (Figure 1). Additionally, information on the top 10 significantly upregulated and downregulated circRNA molecules is listed in Tables 5, 6, respectively.

**TABLE 5:** Top 10 significantly upregulated circRNAs in diseased intervertebral disc tissues compared to degenerated intervertebral disc tissues from patients with spinal tuberculosis.

circRNA	Parental gene	Regulation	FC (abs)	*P*-value	circRNA type	Chrom
hsa_circ_0031968	FERMT2	up	79.2859848	0.000039545	exonic	chr14
hsa_circ_0001806	CSPP1	Up	62.0183803	0.0001233	exonic	chr8
hsa_circ_0077607	FIG4	Up	61.9872718	0.000455687	exonic	chr6
hsa_circ_0071312	KIAA0922	Up	46.9444317	0.00006816613	exonic	chr4
hsa_circ_0062683	TPST2	Up	40.5306465	0.000153663	exonic	chr22
hsa_circ_0000520	RPPH1	Up	38.2061816	0.002526213	sense overlapping	chr14
hsa_circ_0062682	TPST2	Up	36.3737225	0.000142549	exonic	chr22
hsa_circ_0050648	HSPB6	Up	33.2690448	0.00002691171	exonic	chr19
hsa_circ_0008274	UGGT2	Up	33.1685012	0.001022248	exonic	chr13
hsa_circ_0032704	TTLL5	Up	31.0508644	0.000189482	exonic	chr14

**TABLE 6:** Top 10 significantly downregulated circRNAs in diseased intervertebral disc tissues compared to degenerated intervertebral disc tissues from patients with spinal tuberculosis.

circRNA	Parental gene	Regulation	FC (abs)	*P*-value	circRNA type	Chrom
hsa_circ_0004183	FRMD4A	Down	145.5403139	0.01503737	exonic	chr10
hsa_circ_0000367	SIAE	Down	135.1493592	0.005724482	exonic	chr11
hsa_circ_0000691	ZNF646	Down	98.8118683	0.012264111	antisense	chr16
hsa_circ_0006220	TADA2A	Down	95.7205785	0.006234743	exonic	chr17
hsa_circ_0043278	TADA2A	Down	85.2698612	0.010339522	exonic	chr17
hsa_circ_0001666	FAM120B	Down	64.738025	0.026751231	exonic	chr6
hsa_circ_0021647	CAPRIN1	Down	57.2422271	0.024858842	exonic	chr11
hsa_circ_0021652	CAPRIN1	Down	48.3251799	0.030903186	exonic	chr11
hsa_circ_0056558	R3HDM1	Down	46.9086043	0.021166178	exonic	chr2
hsa_circ_0000288	CAPRIN1	Down	37.3992753	0.042937576	exonic	chr11

Figures 1A: Hierarchical clustering analysis. Red indicates upregulated circRNAs (FC > 2), while green represents downregulated circRNAs (FC < −2). C: Degenerative disc tissue samples; T: STB-affected disc tissue samples. Figures 1B: Volcano plot. Red dots denote differentially expressed circRNAs (FC ≥ 2, *P* < 0.05) in STB-affected discs compared to degenerative discs. Green vertical baselines demarcate FC > 2 or FC < −2, and the horizontal green baseline indicates *P* < 0.05. The right side of the plot highlights circRNAs upregulated in STB discs, while the left side shows downregulated circRNAs. Figures 1C: Scatter plot. This plot visually displays circRNAs with FC > 2 and *P* < 0.05 in STB-affected discs versus degenerative discs. The X-axis and Y-axis represent the mean normalized signal values of degenerative discs and STB-affected discs, respectively. The central green line (FC = 1) indicates no difference between groups. circRNAs above the upper green line (FC > 2) or below the lower green line (FC < −2) are considered significantly differentially expressed.

### Types and chromosomal distribution of differentially expressed circRNAs

**FIGURE 2:**
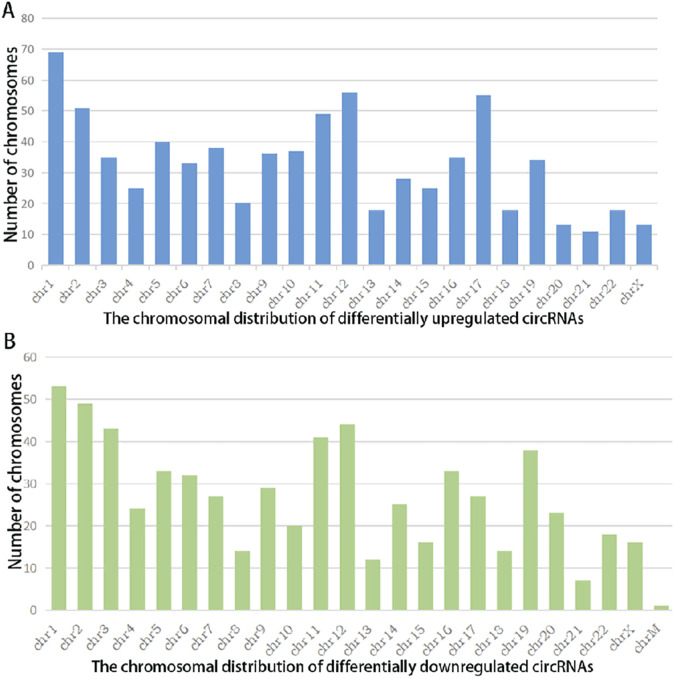
Chromosomal Distribution of Differentially Expressed circRNAs in Spinal Tuberculosis Lesion Tissues Versus Degenerative Disc Tissues: X-axis: Chromosome number; Y-axis: Number of circRNAs mapped to each chromosome; **(A)** Chromosomal distribution of upregulated circRNAs (|FC| ≥2, *P* < 0.05); **(B)** Chromosomal distribution of downregulated circRNAs (|FC| ≤−2, *P* < 0.05).

A summary analysis of significantly differentially expressed circRNAs revealed that among the 757 upregulated circRNAs, 590 (78%) were exonic in type, while among the 639 downregulated circRNAs, 550 (86%) were exonic (Figure 2). This indicates that the majority of differentially expressed circRNAs in the chip expression profile originated from exons, with no significant difference in exonic origin between upregulated and downregulated circRNAs. Further analysis of chromosomal distribution showed that differentially expressed circRNAs were widely distributed across all chromosomes, including the X chromosome. The chromosome with the highest abundance of both upregulated and downregulated circRNAs was chr1, suggesting that circRNAs located on chr1 may play a critical role in the pathogenesis of STB.

#### GO enrichment analysis

GO enrichment analysis was performed on the parent genes of the 1,396 differentially expressed circRNAs (screening criteria: |FC| ≥ 2, *P* < 0.05) to assess their functional annotations in three categories: biological processes (BP), cellular components (CC), and molecular functions (MF).

**FIGURE 3:**
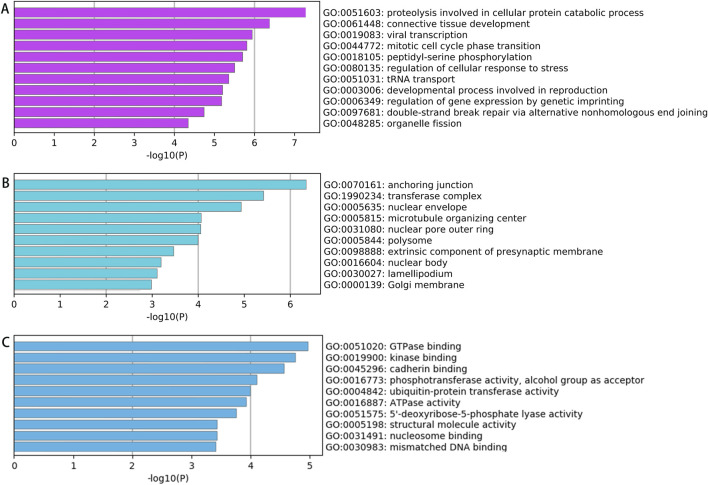
GO Analysis Results of Parent Genes for Significantly Upregulated circRNAs in Diseased vs Degenerated Intervertebral Disc Tissues from Spinal Tuberculosis Patients **(A)** Biological processes; **(B)** Cellular components; **(C)** Molecular functions.

Enrichment Analysis of Upregulated circRNAs. Among the 757 significantly upregulated circRNAs, the top 20 enriched GO terms for their parent genes are shown in Figure 3. Key findings include: BP: Enriched primarily in cellular protein catabolic processes. CC: Enriched in structures such as anchoring junctions, focal adhesions, adherens junctions, cell-substrate junctions, and the actin cytoskeleton. MF: Associated with GTPase binding, enzyme activator activity, GTPase regulator activity, and cadherin binding. These results suggest that upregulated circRNAs may regulate mechanisms linked to protein degradation, cell adhesion, and GTPase signaling in spinal tuberculosis pathogenesis.

**FIGURE 4:**
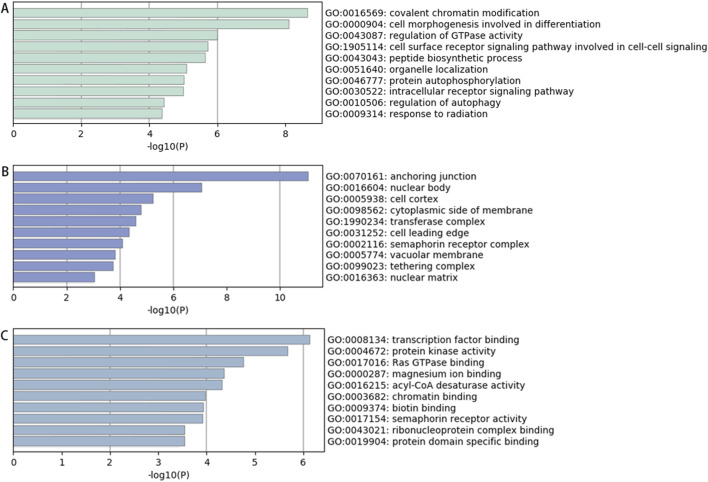
GO Analysis Results of Parent Genes for Significantly Downregulated circRNAs in Diseased vs Degenerated Intervertebral Disc Tissues from Spinal Tuberculosis Patients **(A)** Biological processes; **(B)** Cellular components; **(C)** Molecular functions.

Enrichment Analysis of Downregulated circRNAs. Among the 639 significantly downregulated circRNAs, the top 20 enriched GO terms for their parent genes are shown in Figure 4. Key findings include: BP: Enriched primarily in processes such as covalent chromatin modification, histone modification, peptidyl-lysine modification, and methylation. CC: Enriched in structures including anchoring junctions, adherens junctions, focal adhesions, cell-substrate adhesive junctions, and actomyosin complexes. MF: Associated with transcription factor binding, transcription co-regulator activity, transcriptional coactivator activity, nuclear hormone receptor binding, protein kinase activation, protein serine/threonine kinase activation, and phosphotransferase activity. These results suggest that downregulated circRNAs may influence pathways related to epigenetic regulation (e.g., chromatin/histone modifications) and transcriptional activation in spinal tuberculosis pathogenesis.

### KEGG signaling pathway analysis results

**FIGURE 5:**
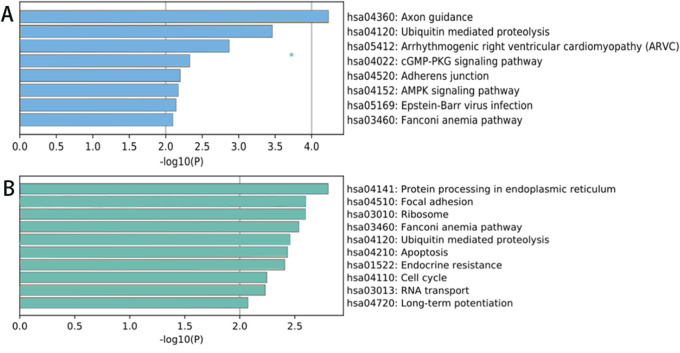
KEGG signaling pathway analysis of circRNA parental genes showing significant differences between lesion and degenerated intervertebral disc tissues from spinal tuberculosis patients. **(A)** Downregulated; **(B)** Upregulated.

The results of KEGG signaling pathway analysis indicate that the parental genes of the upregulated circRNAs are mainly enriched in processes such as protein processing in the endoplasmic reticulum, focal adhesion, ribosome, Fanconi anemia pathway, ubiquitin-mediated protein degradation pathway, apoptosis, endocrine resistance, cell cycle, RNA transport, long-term potentiation, and amphetamine addiction. The results are shown in Figures 5B.

The parental genes of significantly downregulated circRNAs are mainly enriched in pathways such as axon-mediated protein hydrolysis, cGMP-PKG signaling pathway, calcium signaling pathway, adhesion junction, the AMP-activated protein kinase (AMPK) signaling pathway, the EB virus infection, the Fanconi anemia pathway, etc. The results are shown in Figures 5A.

### The verification results of CircRNA in the peripheral blood of patients with spinal tuberculosis

**FIGURE 6:**
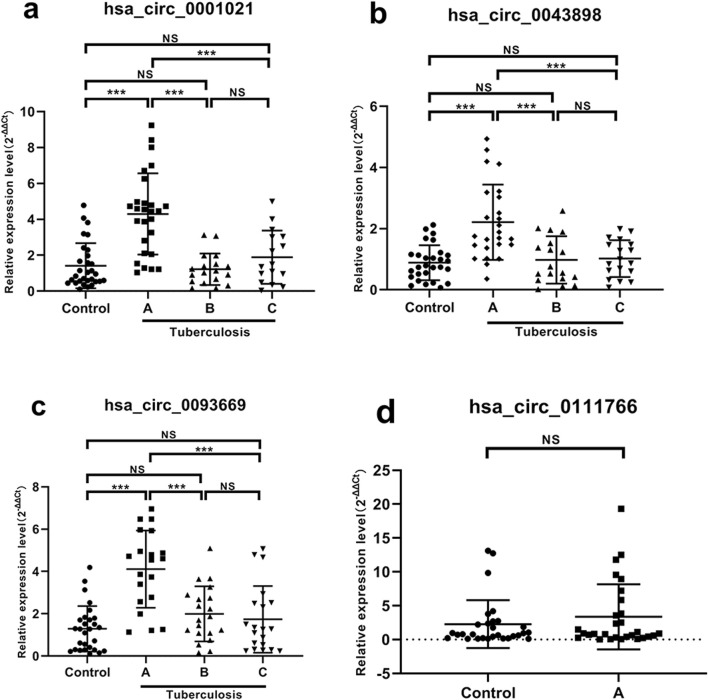
Expression results of each target gene in peripheral blood. Note: **(a)** hsa_circ_0001021; **(b)** hsa_circ_0043898; **(c)** hsa_circ_0093669; **(d)** hsa_circ_0111766. A: Before surgery; B: 6 months after surgery; C: 1 year after surgery; NS *P* > 0.05; ***P < 0.001.

Comparison of expression levels of hsa_circ_0001021 (Figure 6a): Group A showed significantly higher expression compared to the control group, with a statistically significant difference (*P* < 0.001). There was no significant change in Groups B and C compared to the control group, and no statistical difference was observed (Group B: P = 0.978, Group C: *P* = 0.772). Group B and Group C showed significantly lower expression compared to Group A, with statistically significant differences (*P* values <0.001 for both). There was no statistical difference between Group C and Group B (*P* = 0.620). STB patients had stable and high expression of hsa_circ_0001021, hsa_circ_0043898, and hsa_circ_0093669 in peripheral blood compared to the control group. Their expression levels decreased significantly at 6 months and 1 year after postoperative anti-tuberculosis drug treatment, and the differences were statistically significant.

### Correlation analysis between circular RNA expression and inflammatory indicators ESR, CRP, and monocyte/macrophage counts in patients with STB

**FIGURE 7:**
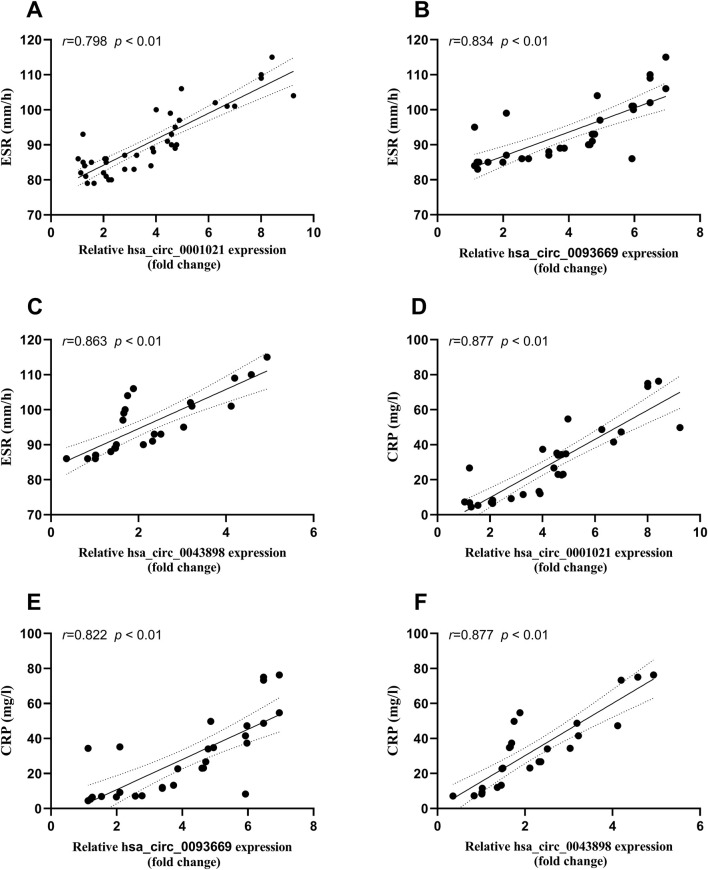
**(A–C)** Correlation between the expression levels of hsa_circ_0001021, hsa_circ_0043898, and hsa_circ_0093669 and ESR (0–20 mm/h) in the observation group; **(D–F)** Correlation between the expression levels of hsa_circ_0001021, hsa_circ_0043898, and hsa_circ_0093669 and CRP (<5 mg/L) in the observation group.

**FIGURE 8:**
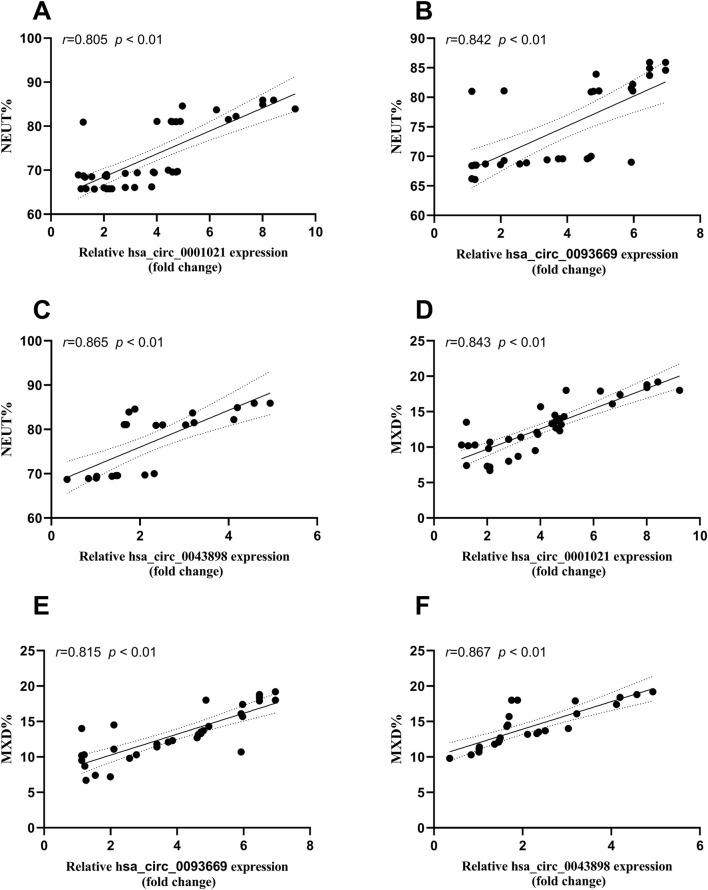
**(A–C)** Correlation between the expression levels of hsa_circ_0001021, hsa_circ_0043898, and hsa_circ_0093669 and the relative value of neutrophils% (NEUT%, 40%–75%) in the blood routine indicators of the observation group; **(D–F)** Correlation between the expression levels of hsa_circ_0001021, hsa_circ_0043898, and hsa_circ_0093669 and the relative value of monocytes% (MON%, 3%–10%) in the blood routine indicators of the observation group.

Bivariate Spearman correlation analysis showed that the relative expression of hsa_circ_0001021, hsa_circ_0043898, and hsa_circ_0093669 in peripheral blood was significantly positively correlated with the levels of inflammatory indicators CRP and ESR (*P* < 0.01), as shown in Figure 7. When MTB enters the patient’s body through the respiratory tract, broken skin, or digestive tract, mononuclear macrophages, as the most important immune effector cells in the human body with strong phagocytic ability, serve as the first important line of defense against MTB entering the body. To further explore this issue, we collected the preoperative blood routine test results of the observation group of STB patients and found that the expression levels of hsa_circ_0001021, hsa_circ_0043898, and hsa_circ_0093669 were significantly positively correlated with the relative values of neutrophils and monocytes (*P* < 0.01), as shown in Figure 8. We summarize the above results and list them in Table 7. These results further confirmed the tissue specificity and potential as molecular markers of hsa_circ_0001021, hsa_circ_0043898, and hsa_circ_0093669 in the circulatory system. Meanwhile, the correlation of hsa_circ_0001021, hsa_circ_0043898, and hsa_circ_0093669 with macrophages suggests that they may play an important role in macrophages.

**TABLE 7:** Correlation analysis between circRNAs and qualitative data of STB patients.

Spearman Rho/Correlation coefficient	ESR	CRP	NEUT%	MXD%
hsa_circ_0001021	0.798**	0.877**	0.805**	0.843**
hsa_circ_0093669	0.834**	0.822**	0.842**	0.815**
hsa_circ_0043898	0.863**	0.877**	0.865**	0.867**

**P < 0.01.

### ROC curve analysis of hsa_circ_0001021, hsa_circ_0043898 and hsa_circ_0093669 in the diagnosis of spinal tuberculosis

**FIGURE 9:**
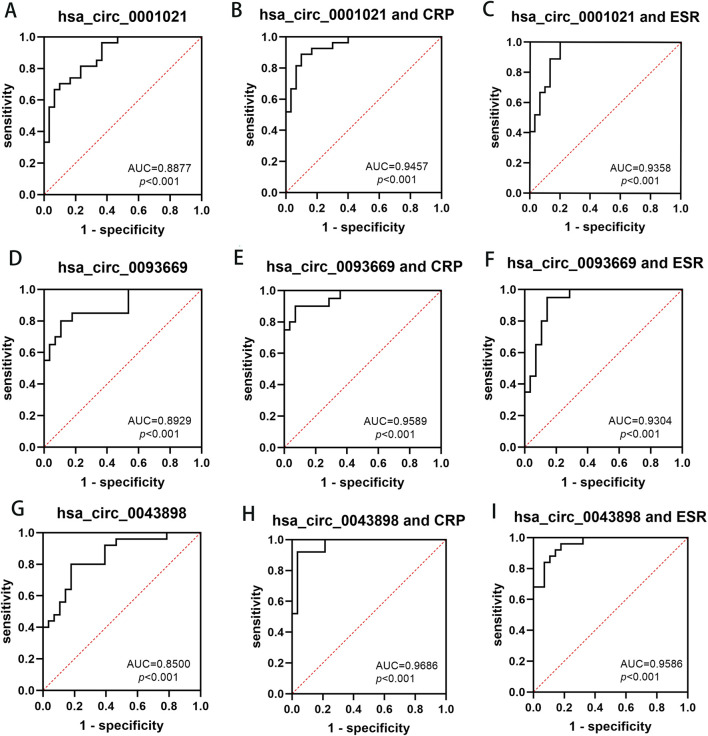
The diagnostic value of hsa_circ_0001021, hsa_circ_0043898, hsa_circ_0093669 in peripheral blood, and their combined use with CRP and ESR for the diagnosis of spinal tuberculosis. **(A)** hsa_circ_0001021; **(B)** hsa_circ_0001021+CRP; **(C)** hsa_circ_0001021+ESR; **(D)** hsa_circ_0093669; **(E)** hsa_circ_0093669+CRP; **(F)** hsa_circ_0093669+ESR; **(G)** hsa_circ_0043898; **(H)** hsa_circ_0043898+CRP; **(I)** hsa_circ_0043898+ESR.

Based on the previous research findings that hsa_circ_0001021, hsa_circ_0043898 and hsa_circ_0093669 were all stably and highly expressed in the peripheral blood of STB patients and showed statistical significance, in order to further study their potential diagnostic value for STB patients, ROC curve analysis was conducted on the data, and the diagnostic value of these three indicators in the peripheral blood of STB patients was further explored, as shown in Figure 9. ESR and CRP are the most commonly used indicators in clinical work to assess inflammation. In STB, they can not only evaluate the degree of infection but also assess the therapeutic effect of the disease. Therefore, the diagnostic value of the combined analysis of these three indicators with ESR and CRP in STB was analyzed separately. We aggregate the above results in Table 8.

**TABLE 8:** ROC Curve Analysis of hsa_circ_0001021, hsa_circ_0043898 and hsa_circ_0093669 in the Diagnosis of Spinal Tuberculosis.

Biomarker	AUC	95*%*CI	*P*	Cut-off value	Sensitivity (%)	Specificity (%)
hsa_circ_0001021	0.888	0.776–0.956	<0.01	0.604	70.37	90.00
hsa_circ_0001021+ESR	0.936	0.838–0.984	<0.01	0.800	100.00	80.00
hsa_circ_0001021+CRP	0.946	0.851–0.988	<0.01	0.789	88.89	90.00
hsa_circ_0043898	0.850	0.725–0.933	<0.01	0.624	80.00	82.14
hsa_circ_0043898+ESR	0.959	0.865–0.994	<0.01	0.781	96.00	82.14
hsa_circ_0043898+CRP	0.969	0.880–0.997	<0.01	0.884	92.00	96.43
hsa_circ_0093669	0.893	0.770–0.964	<0.01	0.693	80.00	89.29
hsa_circ_0093669+ESR	0.930	0.818–0.984	<0.01	0.807	95.00	85.71
hsa_circ_0093669+CRP	0.959	0.858–0.995	<0.01	0.829	90.00	98.26

### Analysis of target miRNAs of CircRNA

**FIGURE 10:**
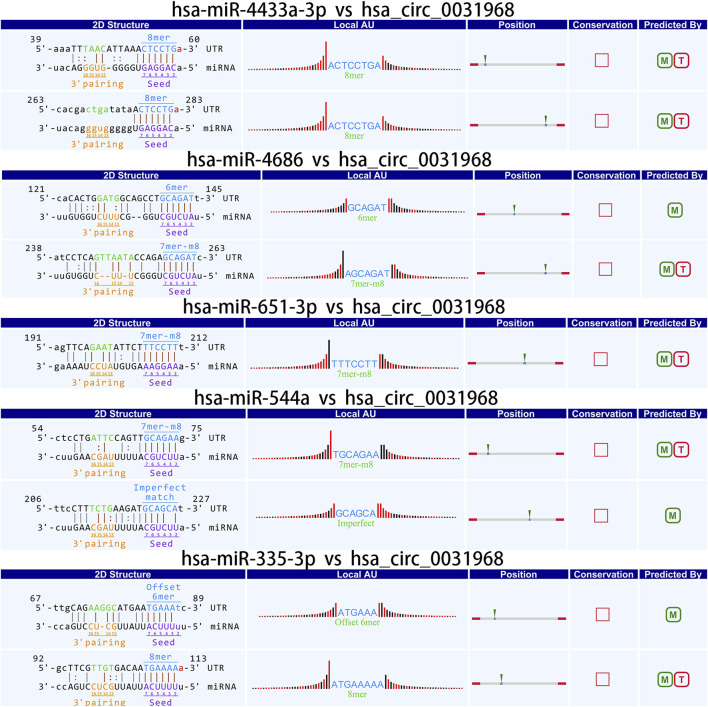
Two-dimensional interaction diagram between hsa_circ_0031968 and its target miRNAs.

**FIGURE 11:**
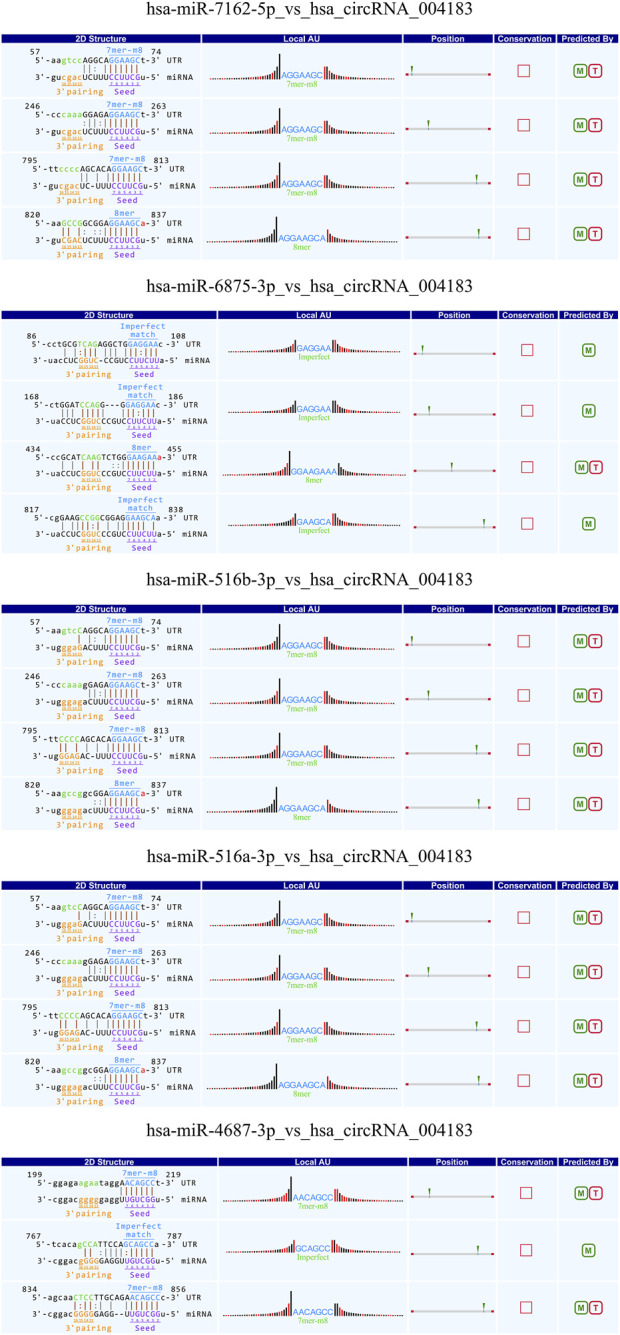
Two-dimensional interaction diagram between hsa_circ_0004183 and its target miRNAs.

Studies have demonstrated that circRNAs reduce miRNA-mediated post-transcriptional inhibition by binding to their target miRNAs through complementary pairing with MREs. To investigate the potential biological functions of these circRNAs, we selected the top ten most significantly upregulated and downregulated circRNAs based on differential expression analysis. By integrating the TargetScan and miRanda algorithms, we predicted miRNAs potentially targeted by these circRNAs. Notably, a single circRNA was found to interact with multiple miRNAs. The top five candidate target miRNAs for each circRNA were prioritized and ranked according to their binding affinity scores and the number of base pairs matched within seed sequences. Furthermore, two-dimensional interaction networks were generated to visualize circRNA-miRNA associations. Representative examples include the network of the upregulated circRNA hsa_circ_0031968 (Figure 10) and the downregulated circRNA hsa_circ_0004183 (Figure 11).

## Discussion

Current research on the pathogenesis of STB remains limited both domestically and internationally. The pathogenesis of STB involves multiple interconnected processes, including macrophage activation, osteoclast differentiation, granulation tissue formation, dysregulation of gene expression, and regulatory imbalances of immune mediators (Naito et al., 2025; Niu et al., 2017). Emerging evidence suggests that STB development results from synergistic interactions among diverse factors, with complex molecular biological underpinnings. Studies investigating STB-associated gene expression have revealed aberrant expression of numerous protein-coding genes during disease progression. Notably, dysregulation of circRNAs—non-coding RNAs with regulatory functions—has garnered increasing attention in recent years, as these molecules may play pivotal roles in modulating critical pathways implicated in STB pathophysiology.

In recent years, the rapid development and widespread application of high-throughput RNA sequencing (RNA-seq) technologies have enabled researchers to identify an increasing number of circRNAs, with growing interest in their mechanistic roles in disease initiation and progression. Studies have revealed that circRNAs are abundant, conserved, stable, and non-random RNA splicing products that regulate gene expression through multiple mechanisms, including miRNA sponging, modulation of parental gene transcription, regulation of cell proliferation, and interactions with RNA-binding proteins (Guo et al., 2020). Notably, circRNAs have been extensively investigated as diagnostic biomarkers and therapeutic targets in cancer biology (Vo et al., 2019; Zheng L. et al., 2022; Zheng R. et al., 2022). However, research on circRNAs in TB remains scarce, with most studies focusing on pulmonary tuberculosis (PTB). These investigations primarily explore differentially expressed circRNAs in peripheral blood as potential diagnostic biomarkers (Huang et al., 2017; Huang Z. K. et al., 2018; Huang Z. et al., 2018; Qian et al., 2018; Fu et al., 2019; Yi et al., 2020), while functional studies elucidating their roles in STB pathogenesis are notably limited (Fu et al., 2019; Yi et al., 2020). Zhang et al. (2017) demonstrated that multiple circRNAs participate in critical pathways in PTB, including endocytosis signaling and mitogen-activated protein kinase (MAPK) signaling cascades. In the context of STB, the only relevant study identified 89 differentially expressed circRNAs in peripheral blood lymphocytes of STB patients using circRNA microarray analysis. Selected circRNAs were validated by qRT-PCR, confirming consistency with microarray results. Subsequent GO enrichment and KEGG pathway analyses revealed that these circRNAs are associated with diverse biological processes and signaling pathways implicated in STB pathogenesis. This study identified differentially expressed circRNAs in the affected intervertebral disc tissues of STB patients and performed GO biological process and KEGG pathway analyses on their host genes. Compared to control samples, 1,396 circRNAs were significantly dysregulated in STB lesions, including 757 upregulated and 639 downregulated species. Most of these circRNAs were exon-derived and broadly distributed across all chromosomes, with chr1 showing the highest circRNA density, suggesting their potential significance in STB pathogenesis. To further investigate the functional implications of these circRNAs in STB pathogenesis, we performed bioinformatics analyses on their host genes. GO analysis revealed enrichment in biological processes such as cellular protein catabolism, GTPase binding, chromatin covalent modification, histone modification, transcription factor binding, transcriptional co-regulator activation, and protein kinase activation. KEGG pathway analysis highlighted key pathways including endoplasmic reticulum protein processing, apoptosis, proteolysis, cGMP-PKG signaling, AMPK signaling, and Epstein-Barr virus (EBV) infection. Notably, prior studies have established mechanistic links between these pathways and TB biology. For instance, filamentous temperature-sensitive protein Z (FtsZ), a GTPase-dependent prokaryotic cell division protein, is inhibited by TB-E12 through suppression of its GTPase activity, thereby restricting MTB growth (Lin et al., 2018). Additionally, GTPase-activating proteins have been implicated in tuberculosis susceptibility (Chen et al., 2019), while kallikrein 12 regulates Mtb-induced autophagy and apoptosis in macrophages via the AMPK pathway (Sabir et al., 2019). Although the EBI3 rs4740 polymorphism is associated with pulmonary tuberculosis susceptibility and EBV infection (Zheng et al., 2015), its relevance to STB remains unexplored. While further experimental validation is required to confirm the role of these pathways in STB development, our findings provide a critical foundation for elucidating molecular mechanisms underlying STB pathogenesis.

STB primarily results from hematogenous dissemination of MTB originating from pulmonary or gastrointestinal foci. Early lesions predominantly localize to vertebral bodies, where terminal arterial vasculature facilitates MTB retention (Kumar et al., 2022). Following initial colonization, MTB may persist in a dormant state when host immunity remains robust. However, systemic immunosuppression or localized trauma can reactivate latent MTB, triggering active STB progression. Diagnosis of STB relies on comprehensive evaluation, including clinical manifestations, imaging studies, laboratory tests, and histopathological confirmation. While experienced clinicians may diagnose typical cases confidently, the majority of STB patients present with insidious onset, nonspecific symptoms (e.g., chronic back pain, low-grade fever), and ambiguous imaging or laboratory findings (e.g., overlapping radiological features with pyogenic spondylitis, variable interferon-gamma release assay sensitivity). These challenges, compounded by limited understanding of STB pathogenesis, frequently lead to delayed diagnosis, misdiagnosis, or inappropriate therapeutic interventions (Sun et al., 2021).

CircRNAs are covalently closed circular structures lacking 5′caps and 3′polyadenylated tails, rendering them resistant to degradation by RNases and RNA exonucleases. In human plasma, circRNAs exhibit an average half-life exceeding 48 hours—significantly longer than the 10-h half-life of linear RNAs (Zhuang et al., 2017). Their enhanced stability, coupled with abundant expression in diverse tissues and biofluids (e.g., blood, plasma, serum, and exosomes) (Geng et al., 2018; Shi et al., 2020), positions circRNAs as ideal candidates for liquid biopsy biomarkers. Recent advances in circRNA research have identified differentially expressed circRNAs in peripheral blood and lesioned tissues of PTB patients, implicating these molecules in disease progression and establishing their utility as diagnostic and prognostic biomarkers (Zhuang et al., 2017; Qian et al., 2018). For instance, Qian et al. (2018) screened peripheral blood mononuclear cells (PBMCs) from active PTB patients and healthy controls, identifying seven significantly dysregulated circRNAs (hsa_circ_0000414, hsa_circ_0000681, hsa_circ_0002113, hsa_circ_0002362, hsa_circ_0002908, hsa_circ_0008797, hsa_circ_0063179), which were validated as potential biomarkers for active PTB. Similarly, Yi et al. (2018) employed circRNA microarrays to detect 75 differentially expressed circRNAs in PTB patient plasma, ultimately validating hsa_circ_103571 as a downregulated diagnostic marker in a cohort of 32 active PTB patients and 29 healthy controls. Collectively, these studies underscore the feasibility of circRNAs as noninvasive biomarkers for tuberculosis. However, current research efforts have predominantly focused on PTB, with limited exploration of circRNA dynamics in STB.

Building upon our initial screening of differentially expressed circRNAs in the intervertebral disc lesions of STB patients, this study further validated their potential as diagnostic biomarkers in peripheral blood. Three circRNAs—hsa_circ_0001021, hsa_circ_0043898, and hsa_circ_0093669—were selected for analysis. Given that ultra-short-course chemotherapy (≤6 months) combined with radical debridement achieves curative outcomes in STB (Yi et al., 2018), we assessed the dynamic expression of these circRNAs in peripheral blood preoperatively, at 6 months postoperatively, and at 1 year postoperatively, alongside inflammatory markers (ESR and CRP). Preoperatively, hsa_circ_0001021, hsa_circ_0043898, and hsa_circ_0093669 exhibited stable and significant upregulation in STB patients compared to controls (*P* < 0.01). ROC curve analysis identified hsa_circ_0093669 as the strongest standalone diagnostic marker (AUC = 0.893, *P* < 0.01; cutoff: 0.693; sensitivity: 80.00%, specificity: 89.29%). While ESR and CRP are established for evaluating infection severity and therapeutic response, multimarker panels enhance diagnostic accuracy (Tahir et al., 2022). Notably, combining hsa_circ_0043898 with ESR yielded optimal diagnostic performance (AUC = 0.959, *P* < 0.01; cutoff: 0.781; sensitivity: 96.00%, specificity: 82.14%), whereas hsa_circ_0043898 paired with CRP demonstrated the highest discriminatory power (AUC = 0.969, *P* < 0.01; cutoff: 0.884; sensitivity: 92.00%, specificity: 96.43%). Longitudinal analysis revealed that circRNA expression levels at 6 months and 1 year postoperatively decreased significantly compared to preoperative levels (*P* < 0.001), reaching parity with controls (*P* > 0.05). No significant differences were observed between 6-month and 1-year postoperative groups (*P* > 0.05), suggesting that these circRNAs not only serve as novel diagnostic biomarkers but also reflect therapeutic efficacy in STB management.

Current research on circRNAs in STB has predominantly focused on their utility as diagnostic biomarkers in peripheral blood, while their functional roles and mechanistic contributions to STB pathogenesis remain largely unexplored. In this study, we conducted the first systematic screening of differentially expressed circRNAs in the intervertebral disc lesions of STB patients, laying a critical foundation for unraveling their involvement in STB development. These findings position circRNAs as promising targets for future mechanistic studies, which may ultimately elucidate their roles in STB pathophysiology. However, challenges persist due to the inherently low expression levels of circRNAs and their primary function as post-transcriptional regulators in target tissues. Notably, this study did not validate the functional relevance of dysregulated circRNAs within lesion tissues, leaving their direct pathogenic contributions to STB unresolved. Further investigations integrating multi-omics approaches and *in vitro*/*in vivo* models are warranted to characterize the molecular networks governed by circRNAs in STB.

This study identified hsa_circ_0001021, hsa_circ_0043898, and hsa_circ_0093669 as significantly upregulated circRNAs in the peripheral blood of STB patients compared to controls, highlighting their potential as novel diagnostic biomarkers. These circRNAs also demonstrated postoperative therapeutic monitoring value, and their diagnostic utility was further enhanced when combined with ESR and CRP levels.

## Limitations

However, a notable limitation arises from the use of patients with lumbar disc degeneration as controls—individuals who are not clinically healthy. Their gene expression profiles and signaling pathways may differ from those of healthy populations, potentially confounding the identification of molecular targets for therapeutic intervention. To address this, future studies will incorporate tissue samples from healthy controls to refine biomarker and target selection. Additionally, *in vivo* validation using animal models will be prioritized to confirm the pathogenic relevance of these circRNAs in STB.

## Conclusion

The differentially expressed circRNAs in the intervertebral disc lesion tissues of STB patients are closely related to the pathogenesis of STB and may become potential targets for the clinical diagnosis or treatment of STB in the future. Hsa_circ_0001021, hsa_circ_0043898, and hsa_circ_0093669 in the peripheral blood of STB patients can serve as biological diagnostic markers. Combined with ESR and CRP, they can significantly improve the diagnostic rate and have certain value in postoperative therapeutic effect evaluation.

## Data Availability

The original contributions presented in the study are publicly available. This data can be found here: https://www.ncbi.nlm.nih.gov/geo/query/acc.cgi?acc=GSE308823.

## Abbreviations

WHO, World Health Organization; STB, Spinal Tuberculosis; MTB, *Mycobacterium tuberculosis*; circRNAs, Circular RNAs; miRNA, microRNA; GO, Gene Ontology; KEGG, Kyoto Encyclopedia of Genes and Genomes; qRT-PCR, Quantitative reverse transcription; BP, biological processes; CC, cellular components; MF, molecular functions; AMPK, AMP-activated protein kinase; CRP, C-reactive protein; ESR, erythrocyte sedimentation rate; PBMCs, peripheral blood mononuclear cells; PTB, pulmonary tuberculosis; FC, fold change; GAPDH, Glyceraldehyde-3-phosphate dehydrogenase.
